# An autopsy case of peripheral T cell lymphoma occurring in a postpartum woman: a unique case suggesting changes in the immunocharacteristics of lymphoma cells before and after delivery

**DOI:** 10.1186/s13000-018-0707-y

**Published:** 2018-05-22

**Authors:** Kenichi Mizutani, Sohsuke Yamada, Xin Guo, Chizuru Futatsuya, Motona Kumagai, Akihiro Shioya, Akane Aikawa, Satoko Nakada, Nozomu Kurose, Hiroshi Minato, Takayuki Nojima

**Affiliations:** 1 Department of Pathology and Laboratory Medicine, Kanazawa Medical University, 1-1 Daigaku, Uchinada, Kahoku, Ishikawa 920-0293 Japan; 2 Department of Pathology, Field of Oncology, Graduate School of Medical and Dental Sciences, Kagoshima University, 8-35-1 Sakuragaoka, Kagoshima, Kagoshima 890-8544 Japan; 3 Department of Pathology, Ishikawa Prefectural Central Hospital, 2-1 Kuratsukihigashi, Kanazawa, Ishikawa 920-8530 Japan; 4 Department of Orthopaedic Surgery, Kanazawa University Hospital, 13-1 Takara-machi, Kanazawa, Ishikawa 920-8641 Japan

**Keywords:** Peripheral T cell lymphoma (PTCL), Autopsy, CD4, CD8, T helper 1 (Th1), T helper 2 (Th2), Pregnancy, Delivery

## Abstract

**Background:**

The occurrence of malignant lymphoma after delivery is an extremely rare event. Although several cases of Hodgkin lymphoma and B cell lymphoma and a few cases of peripheral T cell lymphoma (PTCL) after delivery have been reported, there are no report of autopsy cases of PTCL in the puerperal period.

**Case presentation:**

A 32-year-old Japanese woman with a past medical history of atopic dermatitis and bronchial asthma presented with generalized eruptions four days after the delivery of her first child; generalized skin induration and lymphadenopathy subsequently emerged. A skin biopsy specimen showed the diffuse proliferation of atypical lymphoid cells that were immunohistochemically-positive for CD4 but negative for CD8. She was diagnosed as PTCL, not otherwise specified (PTCL, NOS). She died one year and three months after the onset of symptoms. At autopsy, the systemic infiltration of lymphoma cells into the whole body was observed. Unexpectedly, these lymphoma cells were immuno-reactive with CD8 but not with CD4.

**Conclusion:**

The occurrence and development of PTCL after delivery with the shift from CD4 positivity to CD8 positivity may be associated with not only the selection of resistant subclone as a result of chemotherapy but also the changes of immune status before and after delivery.

## Background

The occurrence of malignant lymphoma after delivery is an extremely rare event. Although several cases of Hodgkin lymphoma and B cell lymphoma of the breast, and one case of PTCL at 3 months after delivery have been reported [1–6], there are no reports of autopsy case of PTCL in the puerperal period. We herein report the first autopsy case of PTCL that occurred exclusively in the puerperal period.

## Case presentation

**Fig. 1:**
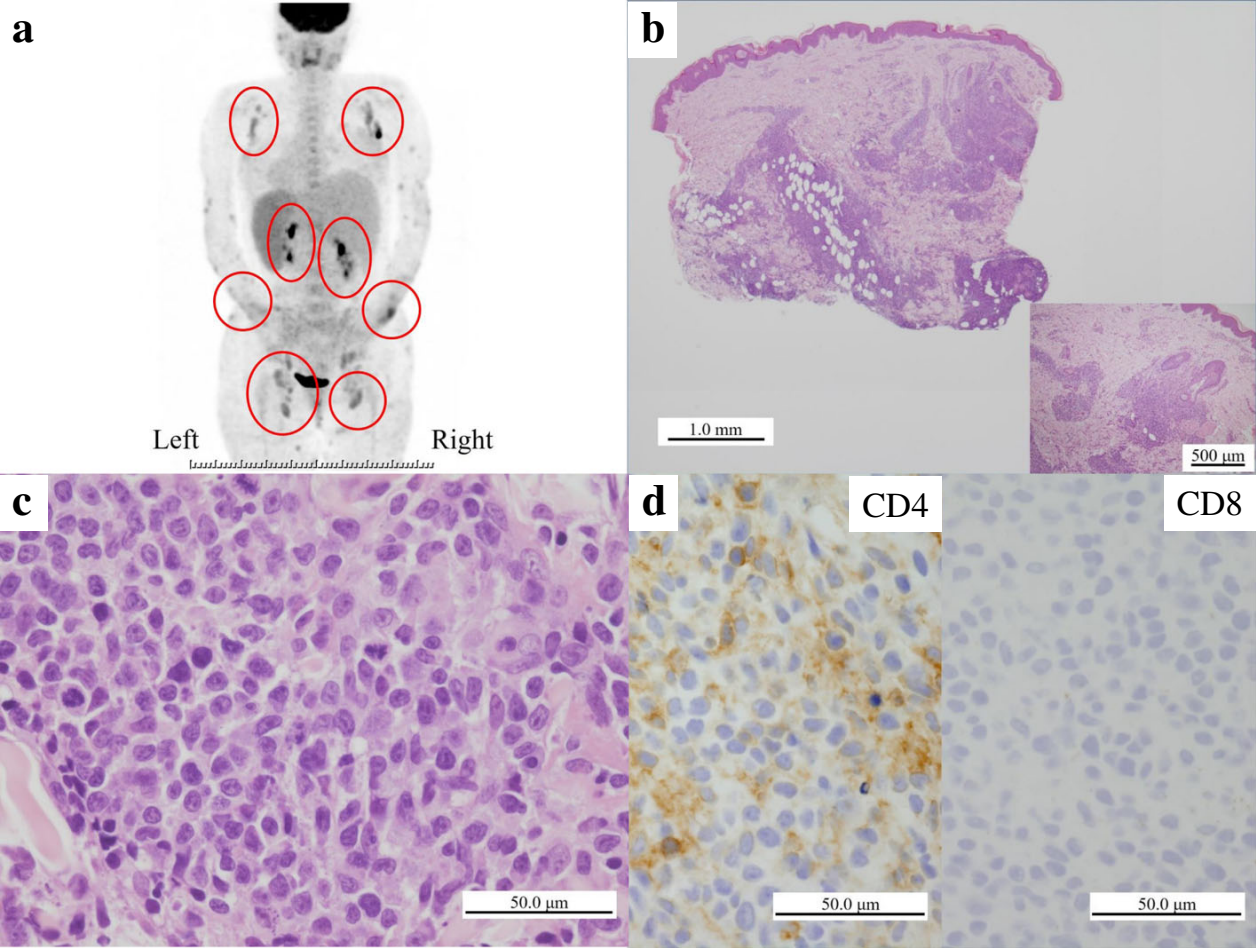
^18^F-FDG-PET, and microscopic and immunohistochemical findings of the skin biopsy specimen. (**a**) ^18^F-FDG accumulated in the axilla, intra-abdominal lymph nodes, inguinal region, and skin of bilateral upper extremities on a whole body ^18^F-FDG-PET scan performed one month after the onset. (**b**) A low-power view of a skin biopsy specimen of the right forearm taken 10 days after the onset of symptoms revealed that lymphoid cells had infiltrated around the vessels and appendages in the middle to deep dermis and subcutis. Bar = 1 mm (H&E staining) (original magnification: × 12.5). Bar = 500 μm (H&E staining) (original magnification: × 40) (inset). (**c**) A high-power view of the specimen revealed mitosis and anisonucleosis of these cells. Bar = 50 μm (H&E staining) (original magnification: × 400). (**d**) Immunohistochemistry revealed that these cells were positive for CD4 but negative for CD8. Bar = 50 μm (original magnification: × 400)

A 32-year-old Japanese woman with past medical history of atopic dermatitis and bronchial asthma presented generalized eruptions four days after the delivery of her first child. Despite administration of topical steroids, generalized skin induration, lymphadenopathy, fatigue, low grade fever and night sweat emerged. A blood test showed leukocytosis with eosinophilia (red blood cell count, 4.31 × 10^6^ /μL; white blood cell count, 10.15 × 10^3^ /μL; neutrophils, 39.3%; lymphocytes, 5.0%; monocytes, 7.9%; eosinophils, 42.4%; basophils, 0.7%; atypical lymphocytes, 0.0%), and a high level of lactate dehydrogenase (LDH; 907 U/L). The patient was negative for HTLV-1 antibodies. An ^18^F-Fluorodeoxyglucose-Positron-Emission Tomographic (FDG-PET) scan revealed the accumulation of FDG in the lymph nodes of whole body and the skin (Fig. 1a). A skin biopsy from the forearm and thigh showed atypical lymphoid cells infiltration around the vessels and appendages in the middle to deep dermis and subcutis (Fig. 1b, c). Although reactive lymphoid cell proliferation and malignant lymphoma were considered as differential diagnosis, lymphoproliferative disorder associated with atopic dermatitis and delivery was initially suspected based on the time course of her problems. T cell receptor gene rearrangement was reported; finally she was diagnosed as PTCL, NOS. Based on immunohistochemistry, these cells were retrospectively considered to be positive for CD4 and negative for CD8 (Fig. 1d). The other immunohistochemistry results were as follows: CD3 and CD5, positive; CD68, Granzyme B and Perforin, focally positive; CD20, CD79a, CD30, CD56, ISH-EBV and TdT, negative. The patient was treated with oral steroids for approximately one month and steroid pulse therapy was administered for three days; however, she experienced repeated relapses and partial responses. Chemotherapies, including CHOEP (Cyclophosphamide, Doxorubisin, Vincristine, Etoposide, Prednisolone), GCD (Gemcitabine, Carboplatin, Dexamethasone), CHASE (Dexamethasone, Cyclophosphamide, Cytarabine, Etoposide, G-CSF), SMILE (Methotrexate, Ifosfamide, Dexamethasone, Etoposide, L-asparaginase), MA (Methotrexate, Ara-C) and Mogamulizumab were attempted; however, the lymphoma relapsed each time. Gradually her condition worsened and she died of the disease at one year and three months after the onset of symptom.

**Fig. 2:**
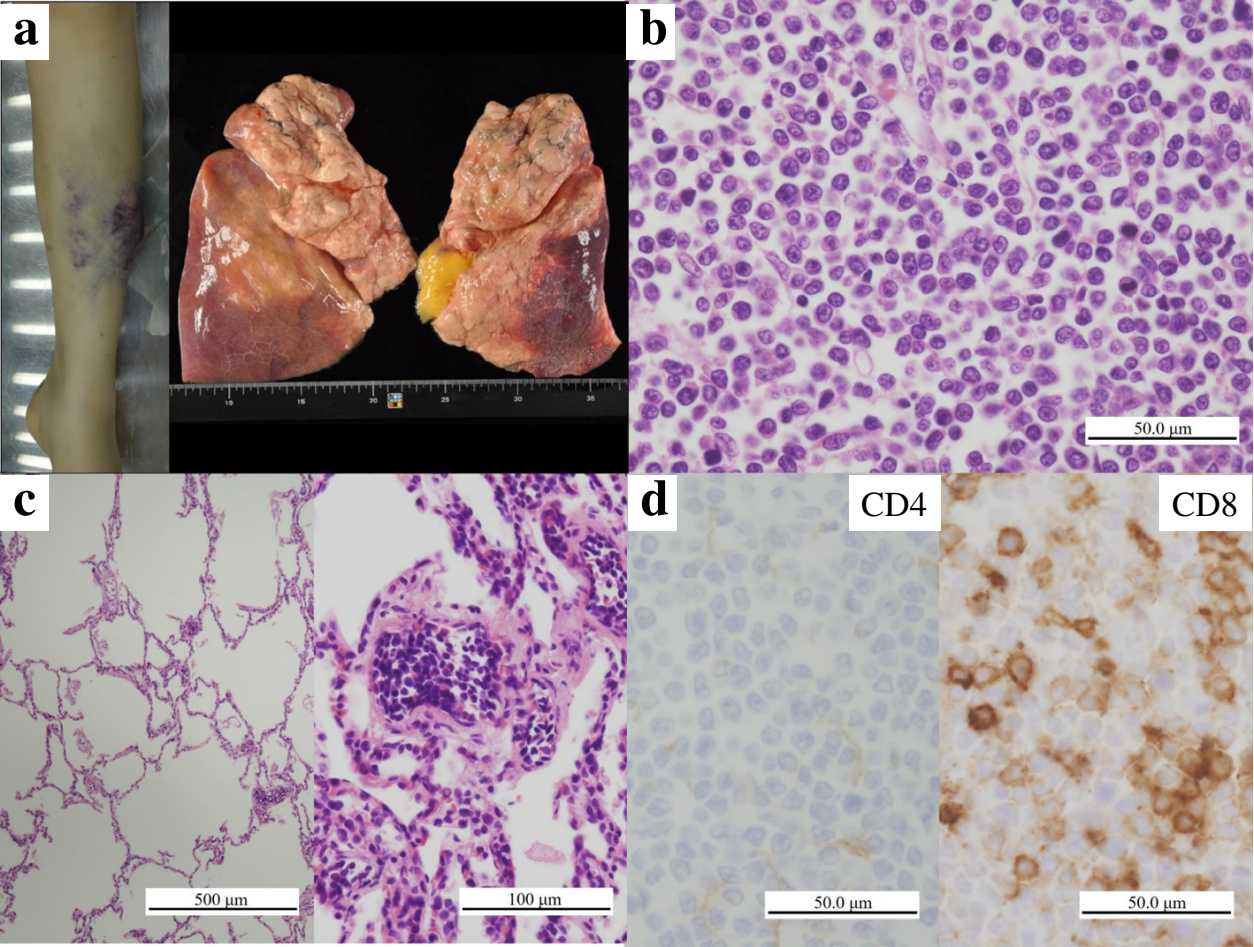
The findings of the skin of lower leg, lung, and right inguinal lymph node at autopsy. (**a**) Purpuric nodules were seen on the bilateral lower leg (left). The lower lobes of the bilateral lungs were congested (right). (**b**) Lymphoma cells with rhomboidal or round nuclei were observed to have diffusely infiltrated into the lymph node. (**c**) Although the alveoli were filled with air (left), the pulmonary alveolar septa and capillary vessels were filled with atypical lymphocytes (right). Bar = 500 μm (H&E staining) (original magnification: × 40) (left). Bar = 100 μm (H&E staining) (original magnification: × 200) (right). (**d**) Immunohistochemistry revealed that the lymphoma cells that had infiltrated the lymph node were negative for CD4 but positive for CD8. Bar = 50 μm (original magnification: × 400)

The patient was 159 cm tall, with a body weight of 61.0 kg; her BMI was 24. Brain autopsy was not performed. At autopsy, multiple purpuric nodules were seen on the body surface (Fig. 2a left). The abdomen was extended with hemorrhagic ascites (2000 ml). A gross examination revealed congestion of the lower lobes of the bilateral lungs (left 255 g, right 435 g) (Fig. 2a right). The spleen, which weighed 830 g, and which had a dark red color was muddy and soft, where hemorrhage was found from the inferior pole. The liver, which weighed 3340 g, had a reddish orange color, was swollen, and multiple small yellow patches were found on its cut surface. The kidney (left 195.6 g, right 164.2 g), had a whitish color, reflecting anemia. Multiple lymph nodes were swollen, including the bronchopulmonary, bilateral inguinal and para-aorta lymph nodes.

Macroscopically, middle-sized lymphoma cells with distinct nucleoli were found to have systemically infiltrated multiple organs, including heart, lung, liver, spleen, pancreas, kidney, breast, adrenal gland, uterus, uterine appendages, lower left leg skin, rib, bone marrow and lymph nodes, including those in the inguinal area (Fig. 2b). The periportal and perivenular nodular infiltration of tumor cells were observed in the fatty liver. In the lungs, alveoli were not broken and filled with air (Fig. 2c left), while capillary vessels of the pulmonary alveolar septa of each lobe were filled with tumor cells (Fig. 2c right). Demyelination was observed in the spinal cord without lymphoma infiltration; however, some lymphoma cells could be found in the vessels. Interestingly, immunohistochemical staining revealed that these lymphoma cells were negative for CD4 but positive for CD8 (Fig. 2d). The other immunohistochemistry results were the same as those of the first skin biopsy: CD5, positive; Granzyme B and Perforin, focally positive; CD20, CD56 and ISH-EBV, negative. Based on these findings, we made a diagnosis of systemic PTCL, NOS, and suggested that the patient’s cause of death might have been respiratory failure because the infiltration of a large number of lymphoma cells in the pulmonary capillaries might have resulted in failed oxygen exchange on the alveolar wall.

## Discussion and conclusion

Pregnancy has some strong effects on the function of immune system, which is closely associated with the risk for malignancy. Several cases of lymphoma after delivery have been reported, most of which were of Hodgkin lymphoma or B cell lymphoma (Table 1). To our knowledge, there are no report of autopsy case of PTCL that occurred in the puerperal period.

**Table 1:** The clinical characteristics of patients with lymphoma after delivery

	Age (years)	Time	Lesion	Disease
[1]	30	During lactation	Bilateral breast	B cell lymphoma
[2]	23	During lactation	Bilateral breast	Burkitt lymphoma
[3]	34	Postpartum	Bilateral breast	Burkitt lymphoma
[4]	Unknown (5 case reports)	During lactation	unknown	Burkitt lymphoma
[5]	Unknown (12 case reports)	Within first 6 months after last delivery	unknown	Hodgkin’s disease
[6]	32	3 months after delivery	Neck, Axilla	ALCL (primary diagnosis) PTCL (recurrent tumor)
Our case	32	4 days after delivery	Heart, Lung, Liver, Spleen, Pancreas, Kidney, Breast, Adrenal gland Esophagus, Stomach, Intestine, Gallbladder, Urinary bladder, Trachea, Thymus, Diaphragm, Peritoneum, Greater omentum, Aorta, Inferior vena cava Bone, Skin, Uterus, Uterine appendages, Lymph nodes	PTCL

**Fig. 3:**
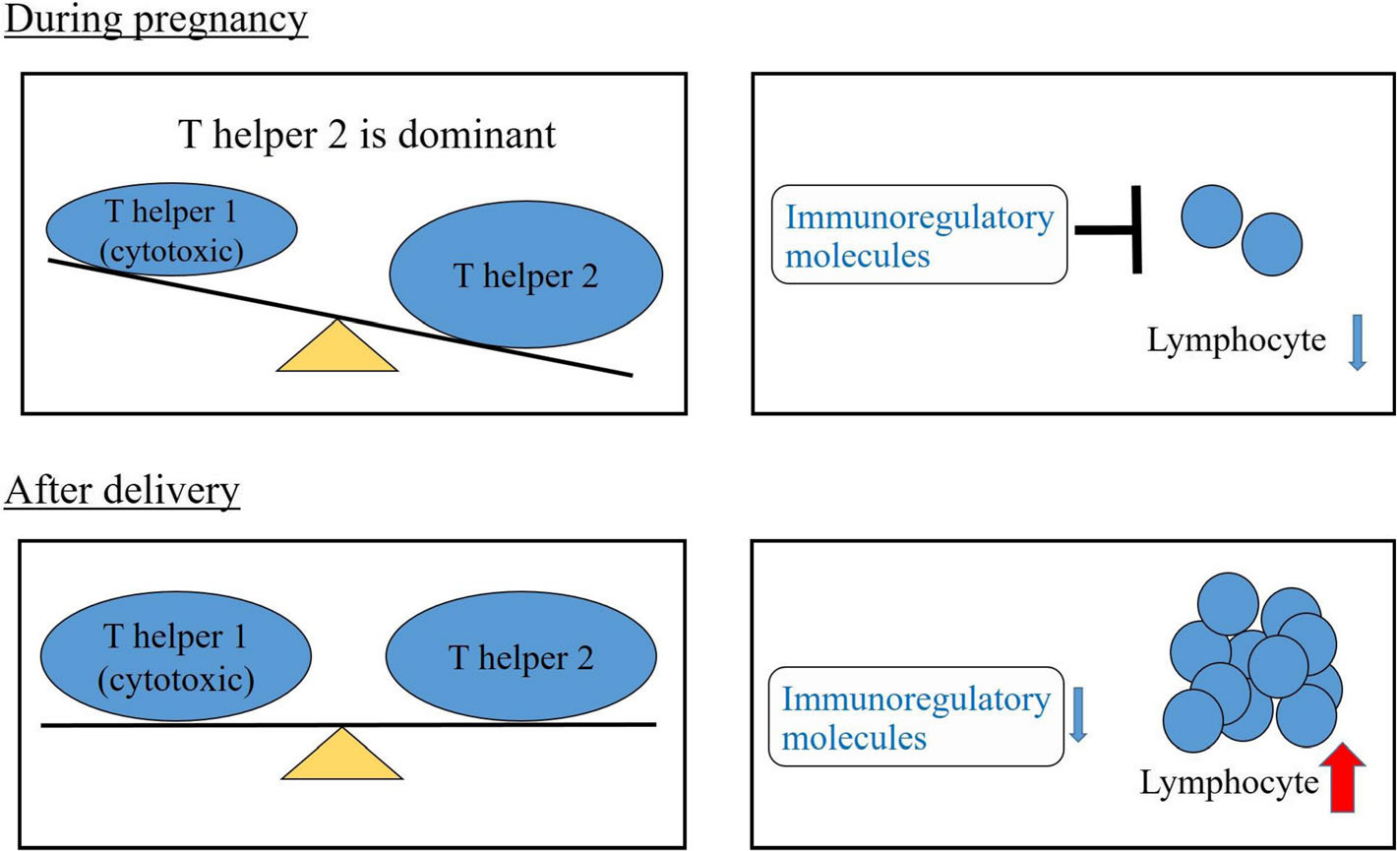
The immune system during pregnancy and after delivery. The T helper 1-type reaction is downregulated during pregnancy. Moreover, immunoregulatory molecules, including progesterone-induced blocking factor (PIBF), placental suppressor factor and trophoblast cell-derived factor, suppress lymphocyte proliferation during pregnancy. After pregnancy, the T helper 2 dominance disappears. In addition, some or all of these immunoregulatory molecules are reduced and lymphocytes can proliferate after delivery

T helper 2 (Th2) dominancy and some immunoregulatory molecules are very important for achieving a successful pregnancy [7]. Th1 and Th2 cells are subsets of CD4-positive T cells. Th1 cells induce cytotoxic and inflammatory reactions which are mediated by some cytokines such as interleukin 2 (IL-2), interferon γ (IFN-γ), tumor necrosis factor β (TNF-β) and IL-12, while Th2 cells secrete IL-4, IL-5, IL-6, IL-9 and IL-10, which are associated with B cell antibody production. Th1-oriented response inhibit Th2-oriented response and vice versa. A study suggested that CD8-positive T cells, which secrete the Th1-type cytokines, were associated with pregnancy loss. Pregnancy is a Th2 dominant situation (Fig. 3). A strong Th2-type reaction tends to suppress Th1-type responses and prevent loss of pregnancy. Furthermore, the expression of some immunoregulatory molecules, such as progesterone-induced blocking factor (PIBF), placental suppressor factor and trophoblast cell-derived factor, which can suppress lymphocyte proliferation, increases during pregnancy (Fig. 3).

CD4-positive T cells, especially Th2 cells, are considered to play an important role in atopic dermatitis (AD)-related inflammation. It was previously reported that the allergen-specific T cells in the blood of patients with chronic atopic dermatitis are mainly associated with the Th2-type response [8].

In this case, although malignant lymphoma was considered as differential diagnosis, reactive lymphocyte proliferation was initially suspected because the onset was just after delivery and the patient had a history of atopic dermatitis. Immunohistochemically, CD4-positive lymphocytes were dominant when the first diagnosis was made, suggesting CD4 predominance before delivery, which was not contradictory to immunocharacteristics of pregnancy or AD. Surprisingly, however, CD8-positive lymphocytes were found to have become dominant in the tumor tissues from autopsy. As stated above, it is suggested that CD8-positive T cells secreting Th1-type cytokines are associated with pregnancy loss and that Th1-type reactions are suppressed during pregnancy. Moreover, lymphocyte proliferation is also prevented. Thus, the proliferation of CD8-positive T cells associated with Th1-type reaction is thought to be suppressed during pregnancy. In this case, the patient’s lymphoma worsened after delivery with a shift from a CD4-dominant state to a CD8-dominant state. Although it is possible that the selection of a resistant subclone occurred as a result of chemotherapy, from the view point of immune system of pregnancy, it is also possible that the immunohistochemical changes and the proliferation of lymphoma cells that were seen in this autopsy case were associated with the delivery. To the best of our knowledge, no studies have reported this feature in PTCL; thus, the present case is very unique.

The systemic infiltration of lymphoma cells was seen at autopsy. The infiltration of lymphoma cells into the capillary vessels of the whole lung may directly lead to the death of the patient. These findings may show the aggressiveness of this lymphoma, which could be another feature of this tumor.

We reported an autopsy case of PTCL that occurred immediately after delivery. In this case, the monotonous proliferation of CD8-positive lymphoma cells may have been activated after delivery; however, further investigation is need to elucidate the detailed relationship between pregnancy, child-birth and PTCL. There was shift in the immunohistochemical characteristics of the tumor cells, from CD4 positivity to CD8 positivity, which may be associated with not only the effect of chemotherapy but also the change before and after delivery. This is the most unique point of this autopsy case.

## Abbreviations

PTCL: peripheral T cell lymphoma
Th1: T helper 1
Th2: T helper 2
LDH: lactate dehydrogenase
FDG-PET: fluorodeoxyglucose-positron-emission tomography
IL: interleukin
IFN: interferon
TNF: tumor necrosis factor
AD: atopic dermatitis
PIBF: progesteron-induced blocking factor

## References

[1] Antoniou SA (2010). Bilateral primary breast lymphoma masquerading as lactating mastitis. Eur J Obstet Gynecol Reprod Biol.

[2] Negahban S (2010). Primary bilateral Burkitt lymphoma of the lactating breast: a case report and review of the literature. Mol Diagn Ther.

[3] Nomizu T (1986). Burkitt’s lymphoma of the bilateral breasts presenting during lactation. Gan No Rinsho.

[4] Durodola JI (1976). Burkitt’S lymphoma presenting during lactation. Int J Gynaecol Obstet.

[5] Zwitter M (1996). A case-control study of Hodgkin’s disease and pregnancy. Br J Cancer.

[6] Miyuki K (2001). Pregnancy-associated cytotoxic lymphoma: a report of 4 cases. Int J Hematol.

[7] Raghupathy R (1997). Th1-type immunity is incompatible with successful pregnancy. Immunol Today.

[8] Szegedi K, et al. House dust mite allergens Der f and Der p induce IL-31 production by blood-derived T cells from atopic dermatitis patients. Exp Dermatol. 2017:1–3. 10.1111/exd.13438 28887844

